# The use of a three-dimensional dynamic arm support prevents the development of muscle fatigue during repetitive manual tasks in healthy individuals

**DOI:** 10.1371/journal.pone.0266390

**Published:** 2022-04-01

**Authors:** Marie-Hélène Lavallée-Bourget, Alexandre Campeau-Lecours, Jean Tittley, Mathieu Bielmann, Laurent J. Bouyer, Jean-Sébastien Roy

**Affiliations:** 1 Center for Interdisciplinary Research in Rehabilitation and Social Integration (Cirris), Québec City, Canada; 2 Faculty of Medicine, Université Laval, Québec City, Canada; 3 Faculty of Science and Engineering, Université Laval, Québec City, Canada; University of Illinois at Urbana-Champaign, UNITED STATES

## Abstract

Work-related upper extremity disorders are costly to society due to resulting medical costs, presenteeism and absenteeism. Although their aetiology is likely multifactorial, physical workplace factors are known to play an important role in their development. Promising options for preventing work-related upper extremity disorders include assistive technologies such as dynamic arm supports designed to follow the movement of the arm while compensating for its weight. The objective of this study was to assess the effects of a dynamic arm support on perceived exertion, muscle activity and movement patterns of the upper limb during repetitive manual tasks in healthy individuals. Thirty healthy right-handed individuals were allocated either a static or a dynamic task to perform with and without a dynamic arm support. During the task, surface electromyographic activity (anterior and middle deltoid, upper trapezius) and upper limb kinematics (elbow, shoulder, sternoclavicular) were measured using surface EMG and inertial sensors. Results showed that the dynamic arm support significantly reduced perceived exertion during the tasks and limited the development of muscular fatigue of the anterior and middle deltoid as demonstrated by EMG signal mean epoch amplitudes and median frequency of the EMG power spectrum. The dynamic arm support also prevented a decrease in shoulder elevation and an increase in total shoulder joint excursion during static and dynamic task, respectively. These results denote the potential benefits of dynamic arm supports in work environments. Further studies should focus on their efficacy, acceptability and implementability in work settings.

## Introduction

Work-related upper extremity disorders (WRUEDs) represent a major economic burden due to the associated medical costs, presenteeism and absenteeism [1]. Although multiple physical workplace risk factors have been identified, the most commons contributing to WRUED are performing repetitive and forceful work, and using sustained non-neutral positions [2]. These factors may lead to muscle fatigue, reduce recovery time [3], and ultimately to the development of maladaptive movement patterns [4]. Performing dynamic tasks above shoulder height is also known to lead to rapid development of fatigue in upper limb muscles and, once movement is performed in a fatigued state, to maladaptive movement patterns [5, 6]. For example, following a standardized shoulder fatigue protocol, decreased glenohumeral elevation and increased trunk and scapular movements were demonstrated during upper limb reaching in elevated arm positions [7]. Such adaptations may lead to higher physical stress on periarticular structures such as tendons and muscles [3, 4], and ultimately to WRUEDs.

In this context, attention has been turned toward the development of assistive technologies. Assistive technologies are commonly used in populations with neuromuscular diseases to improve anti-gravity upper limb movements [8]. Nowadays, applications of these technologies have been broadened to other groups such as militaries and workers [9]. There is a wide diversity of upper limb assistive support devices, ranging from simple armrest to powered exoskeletons [10]. Support devices can be classified by activation method: non actuated devices, passively actuated (e.g.: spring, elastic, counterweights) or actively actuated (e.g.: pneumatic, electric) [10]. Furthermore, support devices specific to joint motion can either be classified as exoskeletons (aligned with joints and attached to at least two segments) and end-effectors (not aligned with joints and attached to a single point) [10]. End-effectors such as passively actuated three-dimensional dynamic arm supports (3D-DASs) are one of the most promising types of assistive technologies for the prevention of WRUEDs. 3D-DASs, designed to follow horizontal and vertical arm movements while compensating the weight of the arm [11], can be used by workers performing repetitive arm elevation or using sustained positions in elevated arm positions. Different types of wrist and arm supports have shown the ability to reduce peak and mean muscle activity during repetitive tasks, to reduce perceived exertion of the upper limb, and to even increase work performance during work-related tasks [12–18]. However, all of the evidence showing the potential of arm supports has been obtained when performing tasks with the arm below 45° of elevation.

Repetitive or sustained overhead activities (above 60° of shoulder elevation) are frequently performed by skilled and underqualified manual workers [19]. These overhead activities are known to be particularly demanding for the shoulder because the stability of the glenohumeral joint have been shown to decrease in elevated positions [20, 21]. The maladaptive movement patterns that may emerge following fatigue [22] could therefore be even more detrimental for the shoulder periarticular structures during overhead activities. Knowing the impact of fatigue on upper extremity muscle activity and movement patterns, the question is could 3D-DASs be used to limit the development of fatigue and maladaptive patterns associated with working in elevated positions?

The effects of 3D-DAS on muscle activity, movement patterns and fatigue during work-related tasks have yet to be formally quantified in elevated arm positions during both static (working in a sustained position) and dynamic (repetitive arm movements) tasks. The objective of the present study was to assess the effect of a 3D-DAS on muscle activity, movement patterns and fatigue of the upper limb during repetitive static and dynamic manual tasks in elevated arm positions in healthy participants. We hypothesized that the 3D-DAS would reduce both the development of muscle fatigue and movement adaptation patterns during static and dynamic tasks.

## Materials and methods

### Participants

Thirty healthy right-handed young adults (16 males and 14 females) were recruited through the institutional mailing list of *Université Laval* and through social media (see Table 1 for participants’ characteristics). Potential participants had no self-reported upper limb or neck pain or movement limitation. They were excluded if they had 1) history of surgery to the upper limb and neck, 2) history of glenohumeral luxation, and 3) previously used 3D-DASs. This project was approved by the institutional review board of the *CIUSSS de la Capitale-Nationale* (#2019–1715), and participants provided written informed consent.

**Table 1:** 

	Static Group	Dynamic Group
**N**	15	15
**Gender**	8M, 7F	8M, 7F
**Age (years)**	26.5 ± 3.2	23.5 ± 1.7
**Height (m)**	1.72 ± 0.08	1.71 ± 0.10
**Body Mass (kg)**	71.2 ± 9.9	72.1 ± 15.3
**Body Mass Index (kg/m** ^**2**^ **)**	24.4 ± 2.7	22.6 ±1.8

### Study design

Participants took part in one 90-minute evaluation session (single day, repeated measures design). Information on sociodemographic status was first collected, followed by the measurement of height and body mass. Participants were then randomly assigned to either a static or a dynamic task using a random number generator and stratification for sex (15 participants performed the static task and 15 others performed the dynamic task). Thereafter, surface electromyographic (sEMG) and inertial measurement unit (IMU) sensors were placed on the right upper limb, and isometric peak torque in shoulder flexion was measured. Each participant then completed their assigned 5-minute task twice, once with and once without the 3D-DAS, with a 15-minute break between the two realizations. The isometric peak torque was reevaluated directly following each task.

### Experimental procedures

Following skin preparation (skin cleaned with alcohol), wireless sEMG sensors (Trigno Wireless EMG system, Delsys, Boston, Massachusetts, USA) were placed on the anterior deltoid, middle deltoid and upper trapezius muscles according to SENIAM guidelines [23]. Then, IMUs (MVN, Xsens Technologies, Enschede, Netherlands) were fixed with loop straps or Velcro tape on the distal forearm, arm, scapula and sternum in accordance with the validated Xsens suggested sensor configuration to allow the simultaneous modelling of elbow, shoulder and sternoclavicular (SC) kinematics [24]. Calibration of IMUs was realized according to manufacturer recommendations before data acquisition. Once the calibration was complete, a signal preview from each sensor was used to verify signal quality. Then, the isometric peak torque was measured during two 5-second contractions in shoulder flexion at 90° of humeral flexion with the elbow extended in a seated position with one-minute breaks between each contraction. Shoulder flexion was selected as the evaluated tasks were mainly performed in the sagittal plane. The isometric peak torque was recorded with a handheld electric dynamometer applied over the distal forearm.

**Fig 1:**
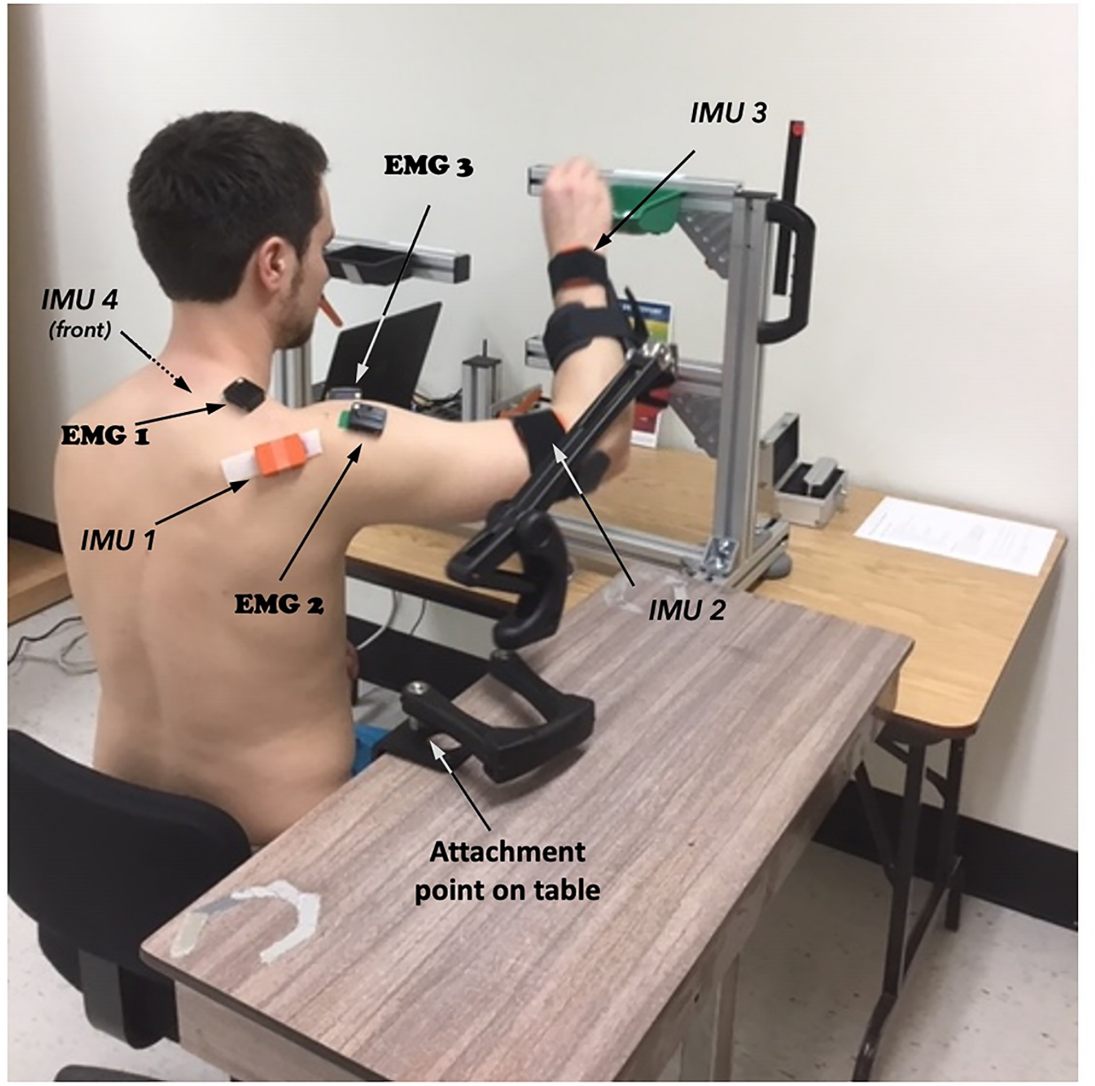
Positions of the three-dimensional dynamic arm support, of the surface electromyographic (EMG) sensors and of the inertial measurement unit (IMU) sensors during the dynamic task. EMG1, upper trapezius; EMG2, middle deltoid; EMG3, anterior deltoid. IMU1, scapula; IMU2, arm; IMU3, distal forearm, IMU4 (not shown on the figure), sternum.

Participants were then seated and an O110 3D-DAS (Kinova, Boisbriand, Quebec, Canada) was installed. The 3D-DAS was fixed on a table with a clamp a few inches behind the back of the chair (Fig 1). The position of the 3D-DAS was adjusted to place the shoulder in neutral position at rest and to allow natural and easy movements in all directions. The O110 3D-DAS is designed to adjust the level of spring compensation easily and to compensate the full weight of the arm using a spring system without restricting arm movements. Twelve levels of spring compensation are available on the O110 3D-DAS (force provided ranges from 8.6 N for level 1 to 55.7 N for level 12), and the level was chosen so that the weight of the arm was fully compensated, without overcompensation. Four participants (two for the static and two for the dynamic task) preferred a spring compensation at level five (force provided of 23.0 N) and all the others were more comfortable at level six (force provided of 26.9 N). Once the 3D-DAS was properly positioned, participants were asked to perform the assigned task for 60 seconds to familiarize themselves with the 3D-DAS and the task. Thereafter, each participant performed the assigned 5-minute task with and without the 3D-DAS. The order was determined randomly, with half of the participants starting without the 3D-DAS, and the other half starting with the 3D-DAS. During the task, sEMG (sampling rate: 1925.93Hz) and kinematic (sampling rate: 60 Hz) data were collected. The two systems were time synchronized using a custom trigger box.

The static task consisted of screwing and unscrewing bolts on rods with the right hand while the arm is at 55° of shoulder flexion. The dynamic task (Fig 1) required participants to move ten bolts, one by one, into boxes placed at different heights using only the right hand. The highest box, at 100° of shoulder flexion, was installed in front of the right shoulder. The bolts then had to be moved down to the second box in line with the first, but at 60° of shoulder flexion. Finally, the third box was in front of the left shoulder at 90° of shoulder flexion. The bolts had to be moved from the first box, to the second, to the third and then back to the first box repeatedly. No speed was imposed for either task, but participants were instructed to execute the task at a natural, self-selected speed in order to reproduce workplace conditions. The number of bolts moved was calculated for each condition. Before and during the task, participants were asked every minute by an automatic vocal recording to rate their level of exertion on the 10-point Borg Rating of Perceived Exertion Scale [25]. To monitor fatigue, the isometric peak torque in shoulder flexion was retested immediately after each condition (with or without arm support) of the task [26].

### Outcome measures and data analysis

#### Perceived exertion

The modified 10-point version of the Borg rating of perceived exertion was used to measure self-reported exertion during the task [25]. The scale ranges from 0 (no exertion at all) to 10 (maximal exertion).

#### Muscle fatigue

Muscle fatigue was indicated by isometric peak torque in shoulder flexion measured before and after each task, as well as sEMG signal mean epoch amplitude and median frequency of the EMG power spectrum (MDF) measured during the tasks. Lower isometric peak torque, higher sEMG mean epoch amplitude and downward shifts in the MDF have been proposed as good indicators of neuromuscular adaptations associated with muscle fatigue [26–29].

*Isometric peak torque*. Isometric peak torque was assessed before and after each task in shoulder flexion using a Medup handheld dynamometer (Atlas-Medic, Quebec City, Canada) as per validated methods [30]. The lever arm was measured using a tape measure from the acromion to the wrist crease. Peak torque value (in Newton-meters) from the two trials was used for statistical analysis.

*sEMG*. All sEMG signals were processed using custom software written in MATLAB R2013a (The MathWorks Inc., Natick, Massachusetts, United States). sEMG signals were digitally filtered off-line with a zero-lag fourth order Butterworth Filter (band-pass 20–450Hz) after mean signal removal. The band-passed signals were rectified, and their sEMG signal amplitude (root-mean-square envelope of the EMG signal) and MDF were first separated in 2-second epochs, and then averaged over periods of 60 seconds for statistical analysis. The power spectral density was computed from a Fast-Fourier Transform, and the MDF quantified using custom software. Downward shifts in the average MDF over periods of 60 seconds during the task were used as an indicator of muscle fatigue. This approach has been shown to work both for static and dynamic movements, regardless of movement velocity [27, 28].

#### Kinematics

All kinematic signals were processed from the raw IMU data using custom software written in MATLAB R2013a. Kinematic signals were digitally low-pass filtered at 8 Hz (Butterworth double-pass filter). The outcomes of interest were joint position during the static task (mean joint position per 60-second period) and total joint excursions and final joint position for the dynamic task. The analyzed joints were the shoulder (elevation), elbow (flexion) and SC joint (elevation). Joint angles at the shoulder were calculated relative to the orientation between the sensors on the sternum and on the arm by applying the ZYZ Euler rotation sequence.

### Sample size and statistical analysis

The sample size, estimated using G * Power 3.1, was based on an average difference of at least 25% between the sEMG signal mean epoch amplitude obtained when performing the tasks with and without 3D-DAS (based on pilot data from our team [unpublished data] and on previous studies evaluating the effects of exoskeletal vests [18, 36]), an alpha error of 0.05 and a power of 0.90. Therefore, 15 participants were needed for each task.

Data were analyzed separately for the static and dynamic tasks. Data distribution was verified using the Shapiro-Wilk test. Paired t-tests were used to compare the maximal level of Perceived Exertion reached on the Borg scale during the task with and without the 3D-DAS, as well as the number of bolts moved during the dynamic task. For isometric peak torque, a one-way repeated-measures ANOVA was used to calculate the effect of Condition (baseline, after the task with 3D-DAS, after the task without the 3D-DAS). For sEMG data and kinematic data during the static task (joint position), a two-way repeated-measures ANOVA was used to compute the effect of Condition (with, without the 3D-DAS) and Time (1^st^, 2^nd^, 3^rd^, 4^th^, 5^th^ minute) and the interaction effect between these factors. For kinematic data during the dynamic task (total joint excursion and final joint position), a three-way repeated-measures ANOVA was used to calculate the effect of Condition (with, without the 3D-DAS), Time (all participants completed the task four times: 1^st^, 2^nd^, 3^rd^, 4^th^ trial) and Target (moving from: 1^st^ box to 2^nd^ box, 2^nd^ box to 3^rd^ box, 3^rd^ box to 1^st^ box) and the interaction effect between these factors; the Target effect and the interaction with the Target were not considered. Inherent post-hoc tests (with Bonferroni adjustment) were conducted in an attempt to detail interactions among factors. To limit the number of post-hoc tests, only comparisons with the 1^st^ minute (sEMG and kinematics during static task; i.e., 1^st^ minute vs. 2^nd^, 3^rd^, 4^th^ and 5^th^ minute) or trial (kinematics during dynamic task; i.e., 1^st^ trial vs. 2^nd^, 3^rd^ and 4^th^ trial) or with the previous minute (i.e., 2^nd^ vs. 3^rd^, 3^rd^ vs. 4^th^, 4^th^ vs. 5^th^) or trial (i.e., 2^nd^ vs. 3^rd^, 3^rd^ vs. 4^th^) were performed. Effect sizes were reported using Partial Eta Squared (η^2^). All statistical tests were conducted in IBM SPSS Statistics (IBM SPSS Statistics 26, IBM Corp., NY, USA) with a significance level of 0.05.

## Results

### Level of perceived exertion and productivity

The mean maximal level of perceived exertion was significantly higher when the task was performed without, compared to with, the 3D-DAS for both the static (p = 0.01 [η^2^ = 0.57]; 1.5 ± 1.0 with, 3.1 ± 1.6 without) and dynamic (p<0.001 [η^2^ = 0.88]; 1.9 ± 1.1 with, 5.2 ± 1.8 without) tasks. While no speed was imposed during the dynamic task, the participants moved the same number of bolts during the 5 minutes of the task with (144±7) and without (148±7) 3D-DAS (p>0.05).

### Fatigue

#### Static task

**Fig 2:**
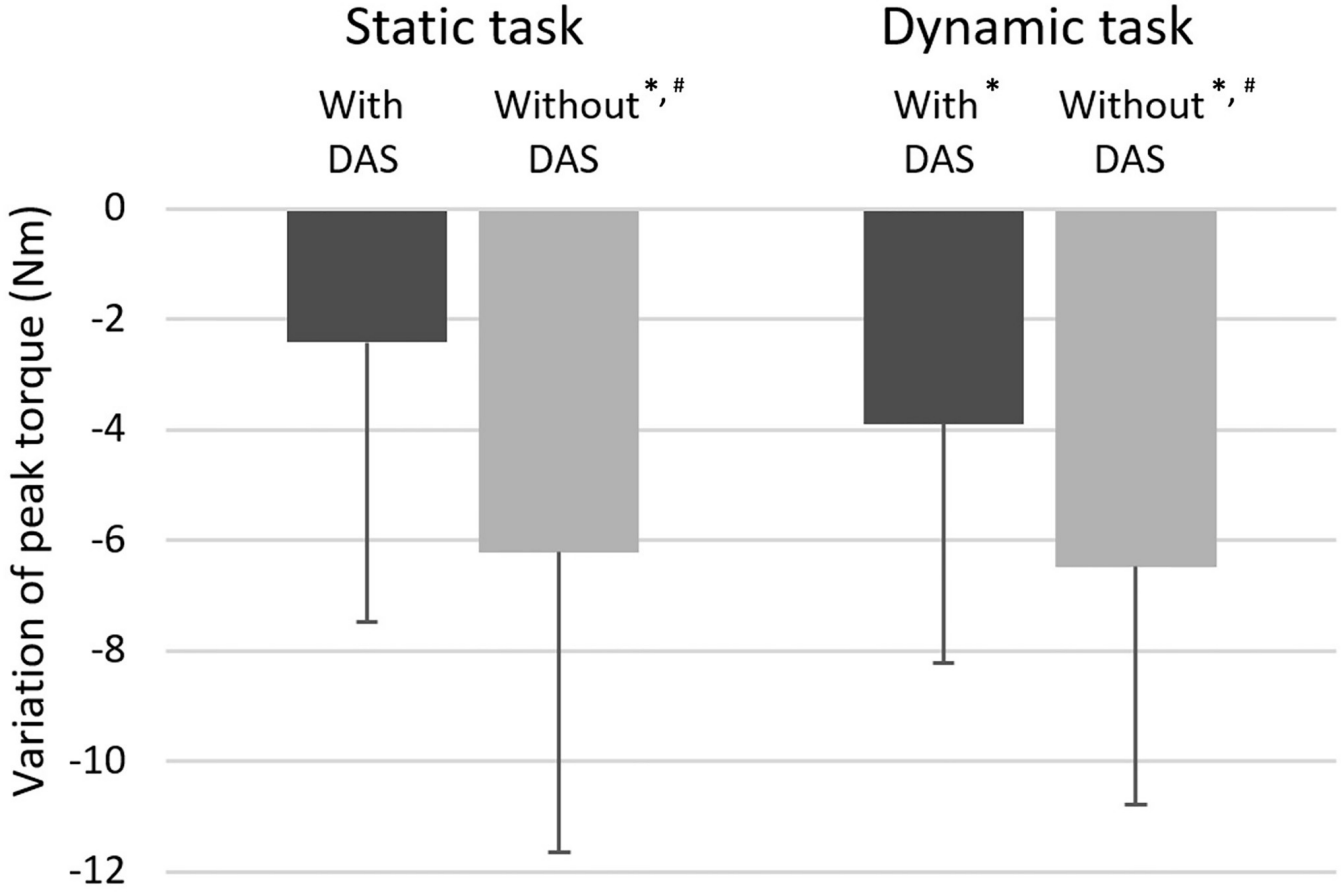
Mean variation in isometric peak torque (in newton-meters [Nm]); With standard deviation) in shoulder flexion after the tasks with and without the dynamic arm support (3D-DAS) when compared to baseline (n = 15 for each task). * Isometric peak torque significantly lower compared to baseline (p<0.003)^#^ Isometric peak torque significantly lower compared to when the task was performed with the 3D-DAS (p<0.015).

A Time effect was observed for isometric peak torque (p<0.001; η^2^ = 0.49). Isometric peak torque was only significantly lower following static task execution without the 3D-DAS when compared to baseline (p<0.001 [η^2^ = 0.61] vs without 3D-DAS; p = 0.059 [η^2^ = 0.23] vs with 3D-DAS). It was also lower after the task without 3D-DAS when compared to with 3D-DAS (p = 0.004; η^2^ = 0.46) (Fig 2).

**Fig 3:**
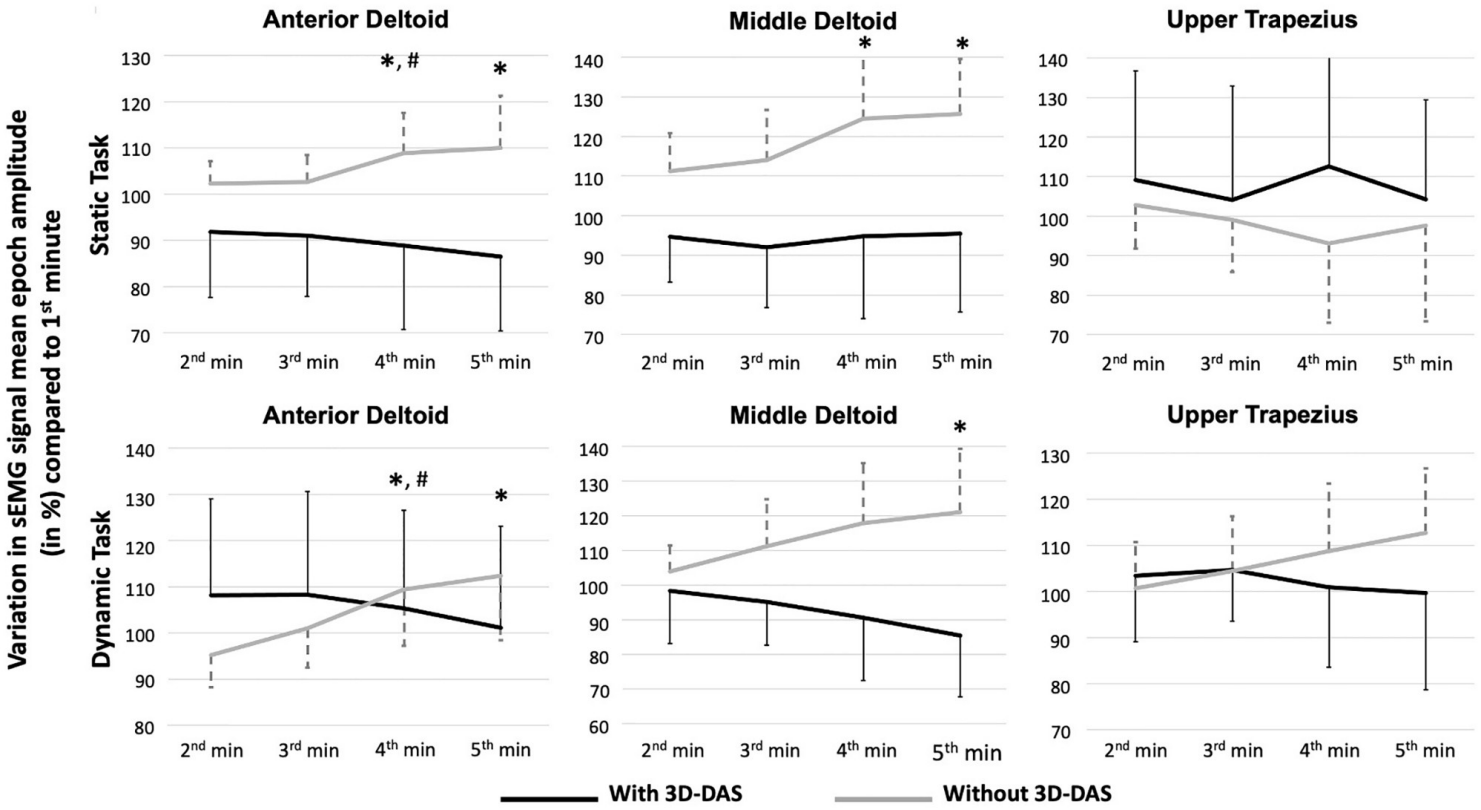
Mean variation in EMG signal mean epoch amplitude (in %; with standard deviation) compared to the first minute during the tasks with and without the dynamic arm support (3D-DAS) (n = 15 for each task). Significantly different (P < 0.05/7 or < 0.007 [Bonferroni adjustment]) compared to *the 1^st^ minute or to ^#^the previous minute when the task was performed without the 3D-DAS.

Regarding sEMG signal mean epoch amplitude, a Condition * Time interaction (p<0.001; η^2^ = 0.44 and 0.44, respectively) and Condition (p<0.001; η^2^ = 0.68 and 0.87, respectively) effects were observed for the middle and anterior deltoid. Posthoc tests for the Condition * Time interaction showed that for both the middle and anterior deltoid, sEMG signal mean epoch amplitude significantly increased when the task was performed without the 3D-DAS (Time effect, p<0.001 for both muscles; η^2^ = 0.44 and 0.45, respectively), while it did not significantly change when performed with the 3D-DAS (Time effect, p>0.119; η^2^ = 0.08 and 0.15, respectively) (Fig 3). For the upper trapezius, only a Condition effect (p = 0.004; η^2^ = 0.48) was observed. The Condition effect for the three muscles showed lower sEMG signal mean epoch amplitude when the task was performed with the 3D-DAS.

**Fig 4:**
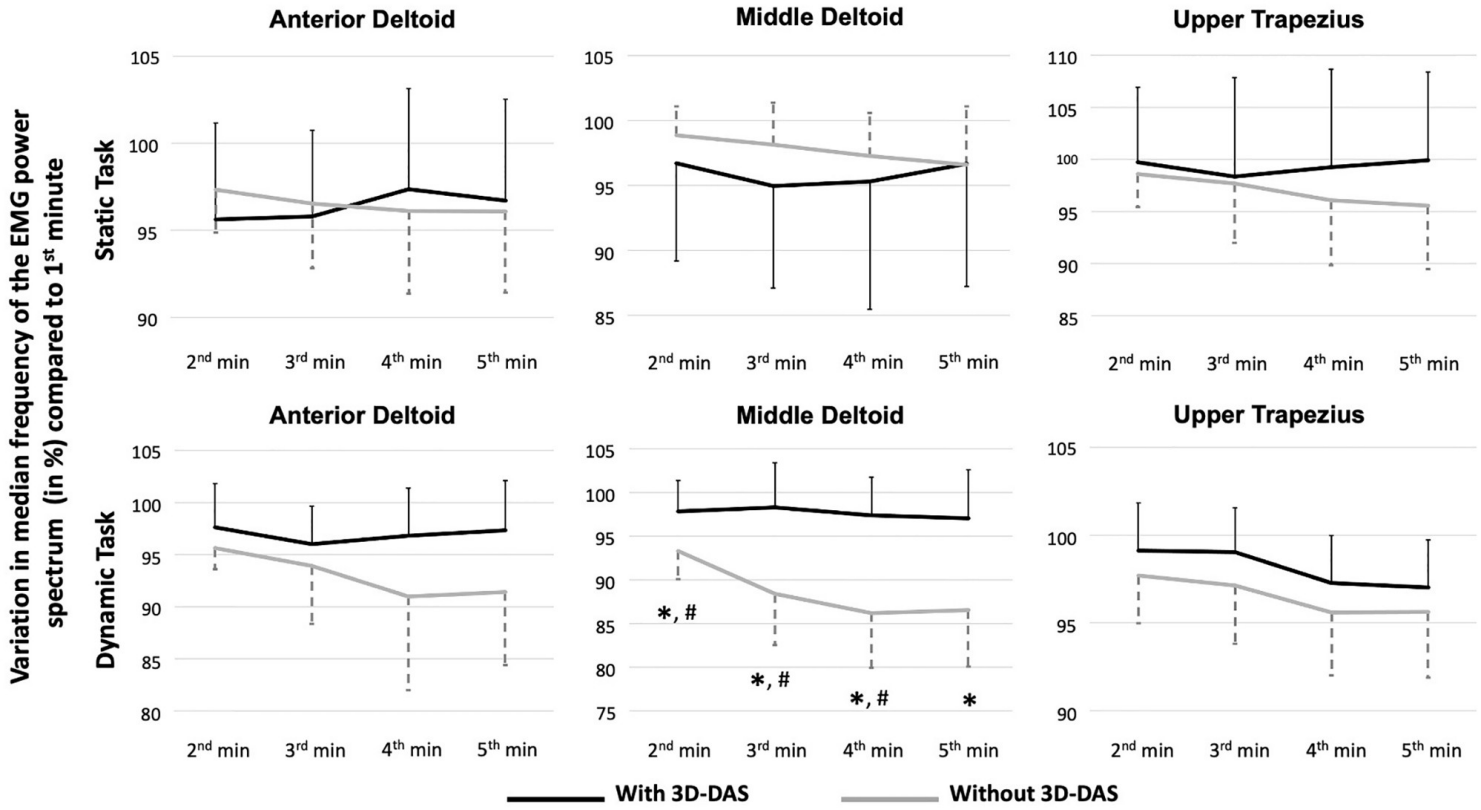
Mean variation in median frequency of the EMG power spectrum (in hertz (Hz); With standard deviation) compared to the first minute during the tasks with and without the dynamic arm support (3D-DAS) (n = 15 for each task). Significantly different (P < 0.05/7 or < 0.007 [Bonferroni adjustment]) compared to *the 1^st^ minute and ^#^the previous minute when the task was performed without the 3D-DAS.

Finally, for the MDF, only a Time effect was observed with a downward shift of MDF during the task for the anterior (p = 0.001; η^2^ = 0.33) and middle (p = 0.026; η^2^ = 0.22) deltoid in both conditions (Fig 4).

#### Dynamic task

Regarding isometric peak torque, a Time effect was observed (p<0.001; η^2^ = 0.63) as isometric peak torque decreased when the task was performed either with (p = 0.003; η^2^ = 0.48) or without (p<0.0001; η^2^ = 0.74) the 3D-DAS when compared to baseline (Fig 2). The decrease in isometric peak torque was also significantly greater when the task was performed without the 3D-DAS than with the 3D-DAS (p = 0.012; η^2^ = 0.45).

For sEMG signal mean epoch amplitude, a Condition * Time interaction (p<0.002; η^2^ = 0.46 for both) and Condition (p<0.0001; η^2^ = 0.64 and 0.77, respectively) effects were observed for the middle and anterior deltoid (Fig 3). Posthoc tests for the Condition * Time interaction showed that for both muscles the mean epoch amplitude significantly increased when the task was performed without the 3D-DAS (Time effect: p<0.001 for both muscles; η^2^ = 0.44 and 0.48, respectively), while it did not significantly change when performed with the 3D-DAS (Time effect: p>0.121; η^2^ = 0.14 and 0.13, respectively). For the upper trapezius, only a Condition effect (p<0.001; η^2^ = 0.85) was observed. The Condition effect for the three muscles showed lower sEMG signal mean epoch amplitudes when the task was performed with the 3D-DAS.

Finally, for MDF, a Condition * Time interaction (p<0.001; η^2^ = 0.68) effect were observed for the middle deltoid (Fig 4). Post-hoc analyses show that the MDF significantly decreased when the task was performed without the 3D-DAS (Time effect: p<0.001; η^2^ = 0.78), while it did not significantly decrease when performed with the 3D-DAS (Time effect: p = 0.07; η^2^ = 0.18). For the anterior deltoid and upper trapezius, only Time effects (p<0.001; η^2^ = 0.56 and 0.57, respectively) were observed. Post-hoc tests show that for both muscles the MDF significantly decreased during the 5-minute task.

### Kinematics

#### Static task

**Fig 5:**
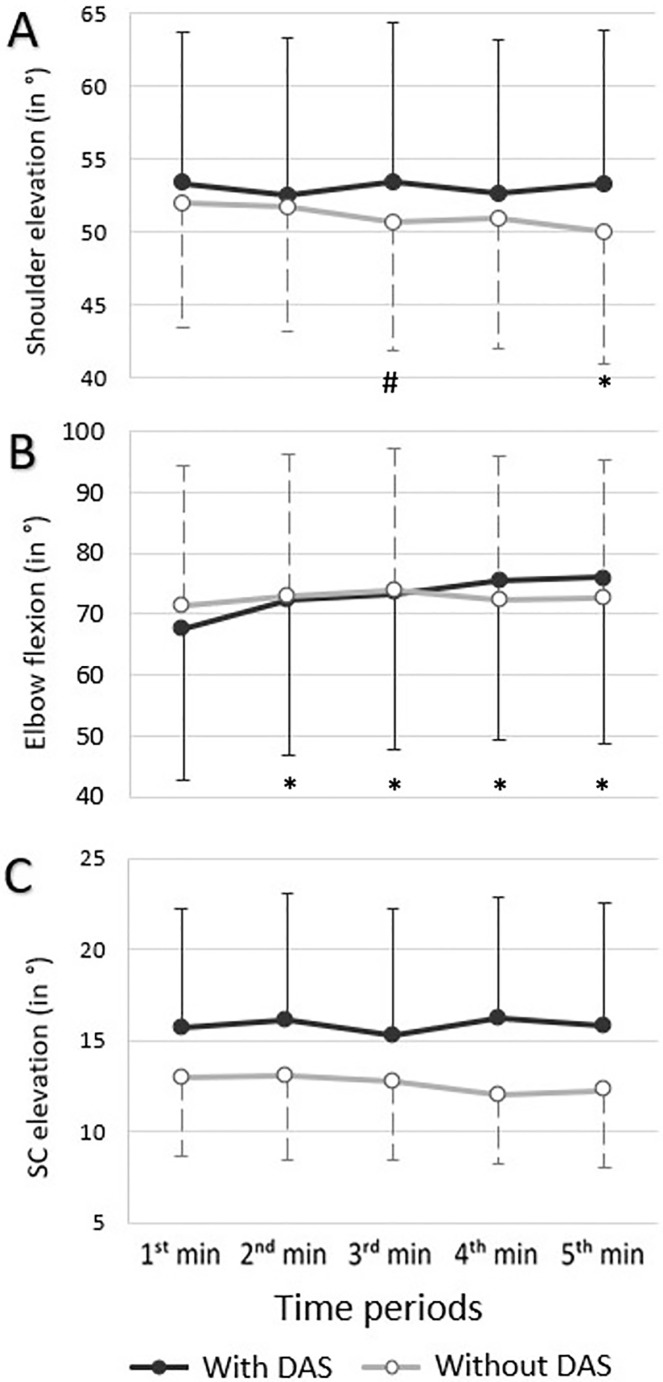
Mean joint position (in degrees; with standard deviation) during the static task in A) shoulder elevation, B) elbow flexion, and C) sternoclavicular (SC) elevation with and without the dynamic arm support (3D-DAS) (n = 15). Significantly different (P < 0.05/7 or < 0.007 [Bonferroni adjustment]) compared to *the 1^st^ minute and ^#^the previous minute when the task was performed without the 3D-DAS for shoulder elevation and when the task was performed with the 3D-DAS for elbow flexion.

A Condition * Time interaction effect was observed for shoulder elevation (p = 0.014; η^2^ = 0.24) and elbow flexion (p = 0.02; η^2^ = 0.37) (Fig 5). For the shoulder, elevation amplitudes significantly decreased when the task was performed without the 3D-DAS (Time effect, p = 0.001; η^2^ = 0.32), while it did not significantly change when performed with the 3D-DAS (Time effect, p = 0.393; η^2^ = 0.07). For the elbow, flexion did not significantly change when the task was performed without the 3D-DAS (Time effect, p = 0.05; η^2^ = 0.18), while elbow flexion significantly increased when the task was performed with the 3D-DAS (Time effect, p<0.001; η^2^ = 0.48). For SC elevation, no Condition * Time interaction, Time or Condition effect was observed (p>0.119).

#### Dynamic task

**Fig 6:**
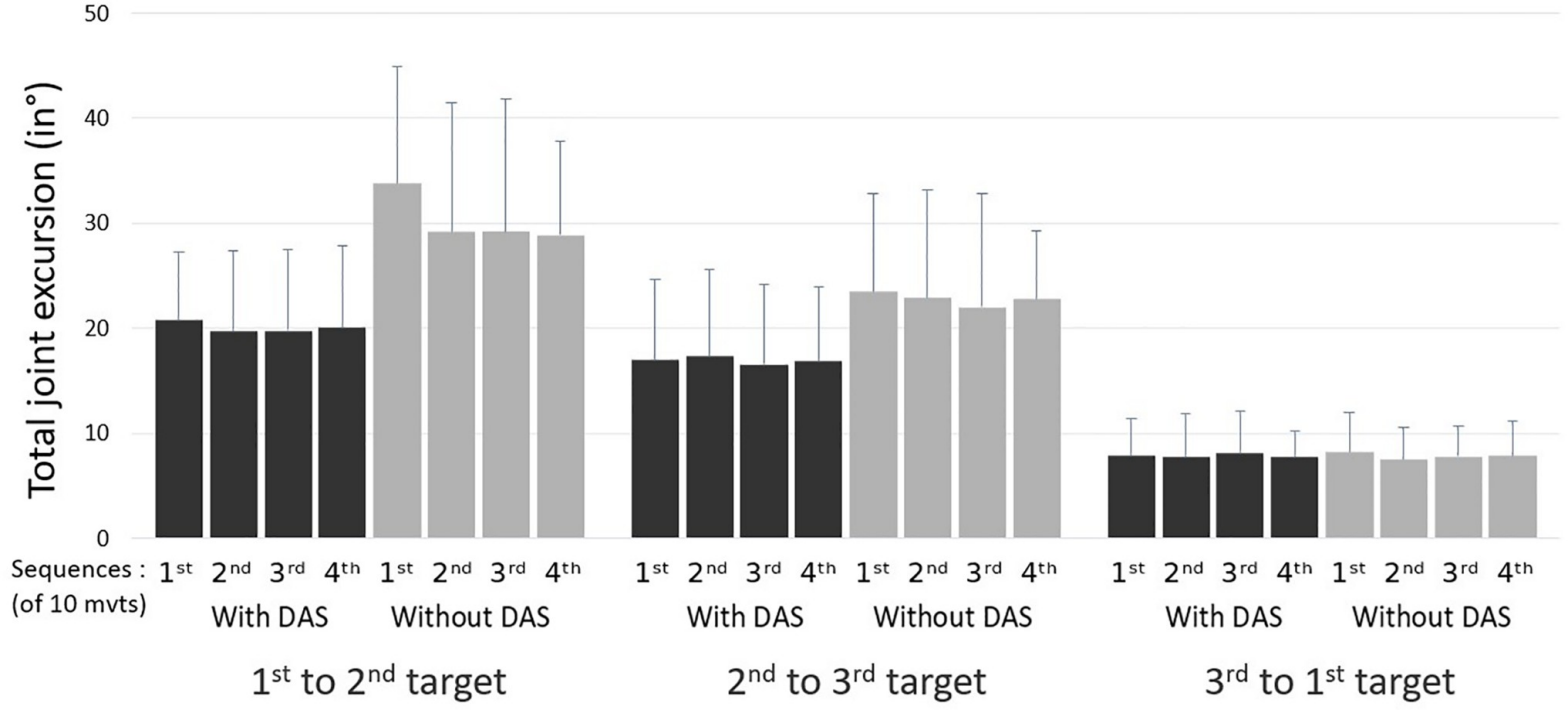
Mean total shoulder elevation excursion (in degrees; with standard deviation) during the dynamic task with and without the dynamic arm support (3D-DAS) (n = 15).

For total joint excursion, only condition effects (p<0.012; η^2^ = 0.42, 0.79 and 0.70, respectively) were observed, showing that total joint excursions in shoulder elevation (Fig 6), elbow flexion and SC elevation were significantly higher when the task was performed without the 3D-DAS compared to with the 3D-DAS.

For the final joint position in shoulder elevation, only a Time effect (p<0.001; η^2^ = 0.55) was observed. Post-hoc tests showed a decrease in shoulder elevation during the task. As for the final joint position in SC elevation, there was a Condition * Time interaction (p = 0.015; η^2^ = 0.39). Post-hoc tests showed that the amplitude in SC elevation significantly increased when the task was performed without the 3D-DSA (Time effect, p = 0.021; η^2^ = 0.37), while it remained stable when performed with the 3D-DAS (Time effect, p = 0.475; η^2^ = 0.05).

## Discussion

The results of this study demonstrate that the use of a 3D-DAS during static and dynamic manual tasks in elevated arm positions reduces perceived exertion, limits the development of muscular fatigue of the anterior and middle deltoid and prevents changes in shoulder kinematics in healthy individuals. Our hypothesis that the 3D-DAS would reduce both the development of muscle fatigue and movement adaptation patterns during static and dynamic tasks was thus confirmed, although the impact of the 3D-DAS was more significant during the dynamic task. Previous studies had already showed that different types of non-actuated or passively actuated wrist and arm supports reduce perceived exertion of the upper limb during typing and pipetting tasks [13, 15], reduce loads on the neck and shoulder muscles during working tasks such as light assembly tasks or keyboard use [12–15] and reduce peak and mean muscle activity during repetitive tasks [16, 18]. Our results are in line with these studies, but differentiate themselves from prior results as we showed a significant and quantifiable effect of the 3D-DAS on muscular fatigue and kinematics for tasks that were performed above 55° and up to 100° of arm elevation, whereas prior studies used 45° of elevation or less.

### Muscular activity

Shoulder elevator muscles have been shown to be the most prone to fatigue during work above shoulder height [31–33]. The deltoid is a very important agonist of shoulder elevation. Its anterior and middle parts progressively contribute to shoulder anterior elevation and abduction in conjunction with the supraspinatus [34, 35]. On the other hand, the upper trapezius helps maintain, along with other scapular stabilisers such as the serratus anterior, the scapula in stable position in order to give a stable base for the rotator cuff muscles to act on and reduce risk of injury of subacromial structures. Its activity has been shown to progressively increase as the arm reach the horizontal [34]. The presence of fatigue in these muscles when performing tasks in elevated arm positions could therefore lead to the development of maladaptive patterns and be detrimental for the shoulder periarticular structures. Our results show that the use of the 3D-DAS reduced sEMG signal mean epoch amplitude in all muscles and minimized the development of muscle fatigue in the deltoid. For example, during the 5^th^ minute of the dynamic task, the sEMG signal mean epoch amplitude was 53% lower for the anterior deltoid, 47% lower for the middle deltoid and 48% lower for the upper trapezius when the dynamic task was performed with the 3D-DAS than when it was performed without. In comparison, other studies obtained similar effects with exoskeletal vests that led to a decrease EMG root mean square in the anterior deltoid of 25 to 55% [18, 36]. The current study shows reduced sEMG activity even at high angles of shoulder elevation, indicating the high degree of support and assistance offered by the 3D-DAS.

In contrast to the middle deltoid, no shift in the MDF was observed in the anterior deltoid and upper trapezius when the dynamic task was performed without the 3D-DAS (Figs 3 and 4). This could be explained by the predominant role of the middle deltoid during this task, as the participants needed a combination of shoulder elevation and abduction to complete the task. The 3D-DAS may thus have offered greater support to the middle deltoid than to the other two muscles. The absence of a significant effect on MDF during the static task may be explained by the fact that dynamic contractions are more effortful than static contractions [5, 37]; thus, the physical demands of the static task may not have been sufficient to lead to a downward shift of the MDF. In fact, in the present study, the sEMG signal mean epoch amplitude during the static task represented 55%, 29% and 35% of the sEMG signal mean epoch amplitude during the dynamic task for the anterior deltoid, middle deltoid and upper trapezius, respectively.

### Movement patterns

In the present study, 3D-DAS significantly prevented the development of adapted movement patterns associated with upper limb fatigue such as decreased glenohumeral elevation and increased scapular movements (Figs 5 and 6). Upper limb fatigue has been shown to lead to reduced joint excursion of the fatigued joint, associated with increased joint excursion of surrounding proximal joints [4, 38]. The upper limb includes multiple joints with closely related functions, which enable it to maintain movement patterns even in a fatigued state [4]. In a fatigued state, an individual may have to increase proximal joints (such as the sternoclavicular joint) and trunk contribution during upper limb movements in order to maintain function [7, 31]. Proximal muscles such as the upper trapezius have been shown to shrug the shoulder girdle, while the trunk muscles bend toward the opposite side to limit the need to elevate the glenohumeral joint when the shoulder elevators are fatigued [31]. These proximal compensations may, however, be a source of injuries [4]. The preservation of shoulder elevation with the 3D-DAS may therefore mitigate the development of adaptative movement patterns that can lead to injuries.

### Workplace implementation

By reducing muscular recruitment and fatigue of shoulder elevators and by limiting the development of adaptative movement patterns of glenohumeral and scapulothoracic joints, 3D-DAS could help prevent WRUEDs for workers who perform repetitive tasks or use sustained positions in elevated arm positions. Before the implementation of the 3D-DAS at work, further validation is needed to first determine its acceptability and implementability in work environments (including its impact on work performance) and whether its use leads to decreased fatigue in work settings. An important consideration in introducing arm supports at work is the necessity of supervision and of monitoring their use. Studies have shown that exoskeletons may increase the risk of injuries, compromise postural control, modify trunk and upper limb kinematics and even increase metabolic demand [39]. As 3D-DASs are usually fixed on a table or a chair, they could lead to unnatural work postures or maladaptive compensations [39] which, if not supervised, could result in an increased risk of injuries [13]. The use of a familiarization protocol, comprising learning sessions about techniques, potential, limits and adjusting of the arm support combined with training scenarios, have been shown to improve user acceptance and performance [40].

### Strengths and limitations

The current study distinguishes itself from prior investigations by including the measurement of both muscular fatigue and kinematics. The impact on these two parameters is central to demonstrating the underlying mechanisms associated with the use of 3D-DASs. The study also quantified 3D-DAS performance in elevated arm positions in both static and dynamic settings, which may support its use in a wider scope of working environments.

There are, however, some limitations. The assessments were performed in a laboratory setting in young and healthy participants using simulated work tasks. As mentioned above, future studies should assess the 3D-DAS in real work environments, but also in older populations and workers. They should also evaluate tasks combining static and dynamic contractions, which are more representative of workers’ tasks. It would have been interesting to evaluate trunk movements, firstly, since trunk involvement can be increased in the presence of fatigue [4], and secondly, because some exoskeletons have been shown to increase the risk of spinal injuries [39]. However, since the 3D-DAS was attached to a table and not supported by the participant as is the case for some exoskeletons, the risk of spinal injuries is reduced since it does not increase spinal loading [41].

sEMG signal amplitude and MDF are good indicators of muscle fatigue and are practical because they are fairly non-invasive [42]. However, these data analysis methods also have their limitations because muscle fatigue is the result of small temporal and spatial changes in muscle fibers that are not necessarily detected by these methods [26, 37] and because they can be influenced by other parameters (e.g., elevation, elevation plane) [43]. sEMG can measure decline in force resulting of many contracting fibers but may not distinguish smaller changes such as dispersion of conduction velocities, action potential amplitude nor metabolic changes in muscular [37]. That being said, sEMG signal amplitude and MDF are still good indicators of muscle fatigue, especially if the analyses focuses on the trend of muscle fatigue (e.g., not for very short periods of time) and for repetitive tasks. Although it was outside of the scope of the present study, measuring antagonist muscle activity during the tasks and the metabolic impact of the 3D-DAS (heart rate or oxygen consumption) would have been interesting as: 1) increased activation of antagonists has been reported with exoskeletons and passive actuated arm supports [6], and 2) some studies have shown a reduction or an increase in metabolic costs (oxygen consumption and heart rate) when using exoskeletons [9, 36, 41, 44].

## Conclusions

Our findings show that the 3D-DAS leads to reduced perceived exertion and muscular load, and to preserved upper limb strength and movement kinematics during static and dynamic tasks. These results denote the potential beneficial effects of 3D-DASs in work environments requiring sustained static or repetitive dynamic upper limb movements. More ecological studies remain necessary before implementing 3D-DASs at work. Still, the promising results of the current study indicate that research should be pursued, as 3D-DASs could help improve working conditions for many workers and reduce the risk of injuries.
